# *N*-(7-Methyl-1,8-naphthyridin-2-yl)acetamide–acetic acid (1/1)

**DOI:** 10.1107/S1600536813005242

**Published:** 2013-03-06

**Authors:** Gao-Zhang Gou, Rui Ma, Qing-Di Zhou, Shao-Ming Chi

**Affiliations:** a College of Chemistry and Chemical Engineering, Yunnan Normal University, Kunming 650500, People’s Republic of China; b School of Computer Science and Technology, Harbin Institute of Technology, Harbin 150001, People’s Republic of China; c School of Chemistry, The University of Sydney, Sydney, NSW 2006, Australia

## Abstract

In the title adduct, C_11_H_11_N_3_O·C_2_H_4_O_2_, all non-H atoms of the acetamide mol­ecule are roughly coplanar, with an r.m.s. deviation of 0.0720 Å. The dihedral angle between the ring plane and the acetamide group is 8.5 (2)°. In the crystal, O—H⋯N and N—H⋯O hydrogen bonds link the acetamide and acetic acid mol­ecules.

## Related literature

For the synthesis of 7-amino-2-methyl-1,8-naphthyridine, see: Brown (1965 ▶); Henry & Hammond (1977 ▶). For the coordination modes of 1,8-naphthyridine ligands, see: Zong *et al.* (2004 ▶); Zúñiga *et al.* (2011 ▶); Li *et al.* (2011 ▶); Gan *et al.* (2011 ▶). For their biological activity, see: Sivakumar *et al.* (2011 ▶); Roma *et al.* (2000 ▶); Badawneh *et al.* (2001 ▶); Nagasawa *et al.* (2011 ▶); Capozzi *et al.* (2012 ▶).
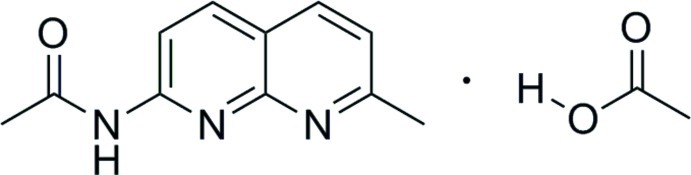


## Experimental

### 

#### Crystal data

C_11_H_11_N_3_O·C_2_H_4_O_2_
*M* *_r_* = 261.28
Triclinic, 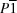

*a* = 8.3628 (17) Å
*b* = 9.0904 (18) Å
*c* = 9.5093 (19) Å
α = 71.30 (3)°
β = 76.43 (3)°
γ = 78.64 (3)°
*V* = 659.8 (2) Å^3^
*Z* = 2
Mo *K*α radiation
μ = 0.10 mm^−1^
*T* = 293 K
0.15 × 0.10 × 0.07 mm

#### Data collection

Rigaku R-AXIS RAPID diffractometer
Absorption correction: multi-scan (*ABSCOR*; Higashi, 1995 ▶) *T*_min_ = 0.986, *T*_max_ = 0.993
5757 measured reflections
2591 independent reflections
1014 reflections with *I* > 2σ(*I*)
*R*_int_ = 0.058

#### Refinement

*R*[*F* ^2^ > 2σ(*F* ^2^)] = 0.047
*wR*(*F* ^2^) = 0.160
*S* = 0.91
2591 reflections
172 parameters
H-atom parameters constrained
Δρ_max_ = 0.21 e Å^−3^
Δρ_min_ = −0.20 e Å^−3^

### 

Data collection: *PROCESS-AUTO* (Rigaku, 1998 ▶); cell refinement: *PROCESS-AUTO*; data reduction: *CrystalStructure* (Rigaku/MSC, 2006 ▶); program(s) used to solve structure: *SHELXS97* (Sheldrick, 2008 ▶); program(s) used to refine structure: *SHELXL97* (Sheldrick, 2008 ▶); molecular graphics: *DIAMOND* (Brandenburg, 1999 ▶); software used to prepare material for publication: *SHELXL97*.

## Supplementary Material

Crystal structure: contains datablock(s) I, global. DOI: 10.1107/S1600536813005242/qm2092sup1.cif

Structure factors: contains datablock(s) I. DOI: 10.1107/S1600536813005242/qm2092Isup2.hkl

Supplementary material file. DOI: 10.1107/S1600536813005242/qm2092Isup3.cml

Additional supplementary materials:  crystallographic information; 3D view; checkCIF report

## Figures and Tables

**Table 1:** Hydrogen-bond geometry (Å, °)

*D*—H⋯*A*	*D*—H	H⋯*A*	*D*⋯*A*	*D*—H⋯*A*
O3—H3*A*⋯N2^i^	0.82	1.96	2.774 (3)	173
N1—H1*A*⋯O2^ii^	0.86	2.07	2.931 (3)	178

Symmetry codes: (i) 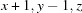; (ii) 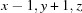.

**Fig. 1.**
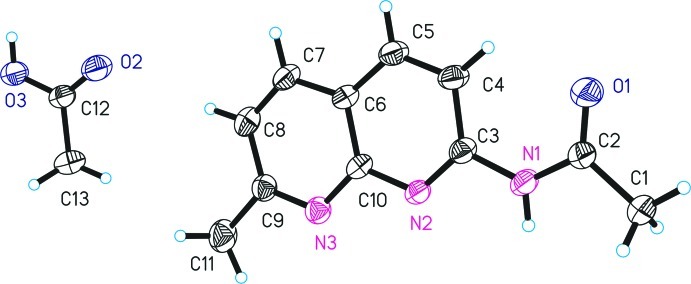
The molecular structure of the title complex with atom labels and 30% probability displacement ellipsoids.

**Fig. 2.**
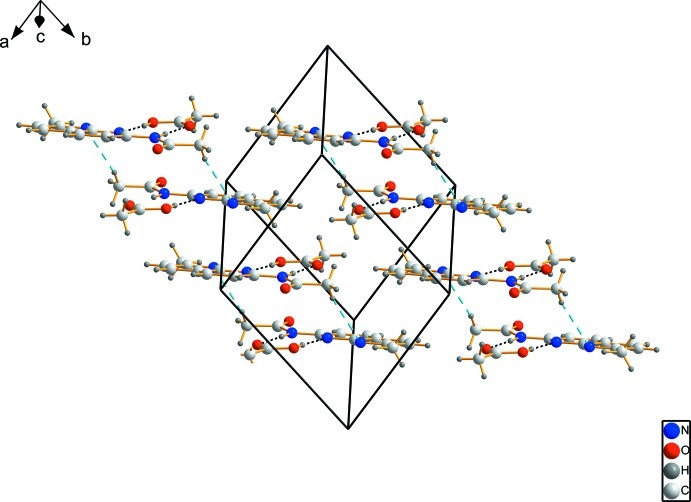
A view of the crystal packing. Hydrogen bonds are shown as black dashed lines, while weak contacts as blue ones.

## supplementary crystallographic information

### Comment

The structure and chemical properties of the 1,8-naphthyridine ring system are interesting to both synthetic and pharmaceutical organic chemists. They can act as monodendate, chelating bidendate and dinuclear bridging ligands(Zong *et al.*, 2004; Zúñiga *et al.*, 2011; Li *et al.*, 2011; Gan *et al.*, 2011). They have also found use as anti-bacterial(Sivakumar *et al.*, 2011), anti-inflammatory(Roma *et al.*, 2000), anti-hypertensive(Badawneh *et al.*, 2001) and anti-cancer drugs(Nagasawa *et al.*, 2011; Capozzi *et al.*, 2012). Herein we report the synthesis and structure of the title co-crystal, C_11_H_11_N_3_O.C_2_H_4_O_2_.

The structure of the title complex is shown in Fig. 1 and Fig. 2 and the hydrogen-bond geometry is given in Table. 1. The planes defined by 7-acetamino-2–methyl-1,8-naphthyridine and acetic acid have root mean square (r.m.s.) deviations of 0.0720 Å and 0.0014 Å and the angle between the planes is 8.66 (19) °. There are two, O—H···N and N—H···O, intermolecular hydrogen bonds between 7-acetamino-2-methyl-1,8-naphthyridine and acetic acid, which link the molecules to form layered units. The O(3)···N(2) and N(1)···O(2) distances are 2.782 (4) and 2.941 (4) Å and the angles O(3)—H(3A)···N(2) and N(1)—H(1A)···O(2) are 173.1 (4)° and 178.0 (2)°. The complementarity of the hydrogen-bonding interactions make the hydrogen-bonded units stable. The stability of these units may explain the difficulty in separating the two components *via* chromatography. The distances between the adjacent parallel planes are 2.960 (4) and 3.349 (4) Å. The weak C—H···N contacts have a H···N distance of 2.666 (3) Å.

### Experimental

7-amino-2-methyl-1,8-naphthyridine(Brown, 1965; Henry & Hammond, 1977)(4.00 g, 0.025 mol) was added to an acetic anhydride (15 ml) solution in an atmosphere of nitrogen. The mixture was stirred at room temperature for 1 h. Followed by slow cooling to room temperature which gave flaky straw-colored crystals. Yield: 3.97 g (78%). In the co-crystal complex, the acetic acid component is formed from the reagent (acetic anhydride) used.

### Refinement

H atoms were placed in calculated positions. The H atoms were constrained to an ideal geometry (C—H =0.96 Å, N—H =0.86 Å and O—H = 0.85 Å) and refined as riding atoms with *U*_iso_(H) = 1.2Ueq(C) or 1.5Ueq(methyl C), *U*_iso_(H) = 1.2Ueq(N) and *U*_iso_(H) = 1.5Ueq(O).

### Crystal data

**Table:**

C_11_H_11_N_3_O·C_2_H_4_O_2_	*Z* = 2
*M**_r_* = 261.28	*F*(000) = 276
Triclinic, *P*1	*D*_x_ = 1.315 Mg m^−^^3^
*a* = 8.3628 (17) Å	Mo *K*α radiation, λ = 0.71073 Å
*b* = 9.0904 (18) Å	Cell parameters from 25 reflections
*c* = 9.5093 (19) Å	θ = 3.1–26.0°
α = 71.30 (3)°	µ = 0.10 mm^−^^1^
β = 76.43 (3)°	*T* = 293 K
γ = 78.64 (3)°	Flaky, yellow
*V* = 659.8 (2) Å^3^	0.15 × 0.10 × 0.07 mm

### Data collection

**Table:**

Rigaku R-AXIS RAPID diffractometer	2591 independent reflections
Radiation source: fine-focus sealed tube	1014 reflections with *I* > 2σ(*I*)
Graphite monochromator	*R*_int_ = 0.058
ω scans	θ_max_ = 26.0°, θ_min_ = 3.1°
Absorption correction: multi-scan (*ABSCOR*; Higashi, 1995)	*h* = −10→10
*T*_min_ = 0.986, *T*_max_ = 0.993	*k* = −11→10
5757 measured reflections	*l* = −11→11

### Refinement

**Table:**

Refinement on *F*^2^	Primary atom site location: structure-invariant direct methods
Least-squares matrix: full	Secondary atom site location: difference Fourier map
*R*[*F*^2^ > 2σ(*F*^2^)] = 0.047	Hydrogen site location: inferred from neighbouring sites
*wR*(*F*^2^) = 0.160	H-atom parameters constrained
*S* = 0.91	*w* = 1/[σ^2^(*F*_o_^2^) + (0.0724*P*)^2^] where *P* = (*F*_o_^2^ + 2*F*_c_^2^)/3
2591 reflections	(Δ/σ)_max_ < 0.001
172 parameters	Δρ_max_ = 0.21 e Å^−^^3^
0 restraints	Δρ_min_ = −0.20 e Å^−^^3^

### Special details

**Table:**

Geometry. All e.s.d.'s (except the e.s.d. in the dihedral angle between two l.s. planes) are estimated using the full covariance matrix. The cell e.s.d.'s are taken into account individually in the estimation of e.s.d.'s in distances, angles and torsion angles; correlations between e.s.d.'s in cell parameters are only used when they are defined by crystal symmetry. An approximate (isotropic) treatment of cell e.s.d.'s is used for estimating e.s.d.'s involving l.s. planes.
Refinement. Refinement of *F*^2^ against ALL reflections. The weighted *R*-factor *wR* and goodness of fit *S* are based on *F*^2^, conventional *R*-factors *R* are based on *F*, with *F* set to zero for negative *F*^2^. The threshold expression of *F*^2^ > σ(*F*^2^) is used only for calculating *R*-factors(gt) *etc*. and is not relevant to the choice of reflections for refinement. *R*-factors based on *F*^2^ are statistically about twice as large as those based on *F*, and *R*- factors based on ALL data will be even larger.

### Fractional atomic coordinates and isotropic or equivalent isotropic displacement parameters (Å^2^)

**Table:**

	*x*	*y*	*z*	*U*_iso_*/*U*_eq_	
N2	0.0316 (3)	0.2121 (2)	0.5279 (2)	0.0452 (6)	
N3	0.2361 (3)	0.1068 (3)	0.3663 (3)	0.0504 (7)	
O3	0.9707 (3)	−0.5868 (2)	0.2515 (2)	0.0681 (7)	
H3A	0.9822	−0.6417	0.3366	0.102*	
C6	0.1937 (4)	−0.0352 (3)	0.6338 (3)	0.0449 (8)	
C10	0.1549 (4)	0.0928 (3)	0.5099 (3)	0.0462 (8)	
C3	−0.0509 (4)	0.2067 (3)	0.6651 (3)	0.0476 (8)	
C4	−0.0218 (4)	0.0819 (3)	0.7965 (3)	0.0551 (9)	
H4A	−0.0838	0.0818	0.8915	0.066*	
O2	0.7624 (3)	−0.4612 (2)	0.3776 (3)	0.0735 (8)	
C5	0.1006 (4)	−0.0375 (3)	0.7772 (3)	0.0541 (9)	
H5A	0.1223	−0.1213	0.8603	0.065*	
N1	−0.1743 (3)	0.3336 (3)	0.6728 (3)	0.0537 (8)	
H1A	−0.1948	0.3949	0.5872	0.064*	
O1	−0.2586 (3)	0.2989 (3)	0.9261 (2)	0.0799 (8)	
C7	0.3229 (4)	−0.1511 (3)	0.6026 (4)	0.0559 (9)	
H7A	0.3533	−0.2369	0.6808	0.067*	
C12	0.8447 (4)	−0.4767 (3)	0.2609 (4)	0.0577 (9)	
C8	0.4028 (4)	−0.1383 (3)	0.4598 (4)	0.0564 (9)	
H8A	0.4878	−0.2153	0.4384	0.068*	
C1	−0.3839 (4)	0.5235 (3)	0.7581 (3)	0.0659 (10)	
H1B	−0.4453	0.5463	0.8492	0.099*	
H1C	−0.4594	0.5117	0.7016	0.099*	
H1D	−0.3215	0.6079	0.6984	0.099*	
C9	0.3559 (4)	−0.0064 (3)	0.3426 (3)	0.0504 (8)	
C11	0.4429 (5)	0.0117 (4)	0.1835 (4)	0.0734 (11)	
H11A	0.3959	0.1073	0.1191	0.110*	
H11B	0.4302	−0.0754	0.1526	0.110*	
H11C	0.5587	0.0150	0.1761	0.110*	
C2	−0.2675 (4)	0.3748 (3)	0.7971 (4)	0.0549 (9)	
C13	0.8161 (5)	−0.3725 (4)	0.1088 (4)	0.0802 (12)	
H13A	0.7230	−0.2939	0.1209	0.120*	
H13B	0.7938	−0.4339	0.0519	0.120*	
H13C	0.9131	−0.3229	0.0561	0.120*	

### Atomic displacement parameters (Å^2^)

**Table:**

	*U*^11^	*U*^22^	*U*^33^	*U*^12^	*U*^13^	*U*^23^
N2	0.0466 (16)	0.0427 (13)	0.0422 (14)	0.0025 (11)	−0.0123 (12)	−0.0088 (10)
N3	0.0497 (17)	0.0534 (14)	0.0479 (15)	−0.0011 (13)	−0.0106 (13)	−0.0165 (11)
O3	0.0696 (17)	0.0648 (13)	0.0544 (14)	0.0067 (12)	−0.0116 (12)	−0.0051 (10)
C6	0.049 (2)	0.0403 (15)	0.0454 (17)	0.0008 (14)	−0.0183 (15)	−0.0089 (13)
C10	0.046 (2)	0.0418 (16)	0.0518 (19)	0.0017 (14)	−0.0195 (16)	−0.0124 (14)
C3	0.050 (2)	0.0474 (16)	0.0463 (18)	0.0025 (14)	−0.0161 (15)	−0.0150 (13)
C4	0.069 (2)	0.0499 (16)	0.0403 (16)	0.0040 (16)	−0.0168 (16)	−0.0073 (13)
O2	0.0814 (19)	0.0733 (15)	0.0530 (15)	0.0150 (13)	−0.0145 (14)	−0.0142 (12)
C5	0.063 (2)	0.0435 (16)	0.0499 (19)	0.0003 (15)	−0.0160 (17)	−0.0061 (13)
N1	0.0588 (19)	0.0493 (13)	0.0455 (14)	0.0120 (13)	−0.0159 (13)	−0.0103 (11)
O1	0.093 (2)	0.0765 (15)	0.0464 (14)	0.0227 (14)	−0.0069 (12)	−0.0092 (11)
C7	0.055 (2)	0.0464 (17)	0.063 (2)	0.0073 (15)	−0.0226 (17)	−0.0115 (14)
C12	0.061 (2)	0.0537 (18)	0.052 (2)	−0.0009 (17)	−0.0167 (18)	−0.0058 (15)
C8	0.054 (2)	0.0514 (17)	0.065 (2)	0.0060 (16)	−0.0167 (18)	−0.0213 (15)
C1	0.062 (2)	0.0562 (19)	0.063 (2)	0.0124 (17)	−0.0070 (18)	−0.0096 (16)
C9	0.047 (2)	0.0543 (18)	0.0519 (19)	−0.0030 (15)	−0.0084 (16)	−0.0209 (15)
C11	0.068 (3)	0.079 (2)	0.070 (2)	0.007 (2)	−0.007 (2)	−0.0303 (18)
C2	0.052 (2)	0.0570 (18)	0.0480 (19)	0.0035 (16)	−0.0085 (16)	−0.0122 (15)
C13	0.083 (3)	0.076 (2)	0.064 (2)	0.000 (2)	−0.024 (2)	0.0049 (18)

### Geometric parameters (Å, º)

**Table:**

N2—C3	1.315 (3)	O1—C2	1.211 (3)
N2—C10	1.365 (3)	C7—C8	1.344 (4)
N3—C9	1.322 (3)	C7—H7A	0.9300
N3—C10	1.353 (3)	C12—C13	1.497 (4)
O3—C12	1.310 (3)	C8—C9	1.413 (4)
O3—H3A	0.8200	C8—H8A	0.9300
C6—C5	1.399 (4)	C1—C2	1.498 (4)
C6—C7	1.402 (4)	C1—H1B	0.9600
C6—C10	1.414 (3)	C1—H1C	0.9600
C3—N1	1.397 (3)	C1—H1D	0.9600
C3—C4	1.426 (4)	C9—C11	1.489 (4)
C4—C5	1.364 (4)	C11—H11A	0.9600
C4—H4A	0.9300	C11—H11B	0.9600
O2—C12	1.195 (3)	C11—H11C	0.9600
C5—H5A	0.9300	C13—H13A	0.9600
N1—C2	1.371 (3)	C13—H13B	0.9600
N1—H1A	0.8600	C13—H13C	0.9600
			
C3—N2—C10	118.3 (2)	C7—C8—C9	119.2 (3)
C9—N3—C10	117.6 (2)	C7—C8—H8A	120.4
C12—O3—H3A	109.5	C9—C8—H8A	120.4
C5—C6—C7	125.0 (3)	C2—C1—H1B	109.5
C5—C6—C10	118.0 (2)	C2—C1—H1C	109.5
C7—C6—C10	117.0 (3)	H1B—C1—H1C	109.5
N3—C10—N2	115.3 (2)	C2—C1—H1D	109.5
N3—C10—C6	123.0 (2)	H1B—C1—H1D	109.5
N2—C10—C6	121.7 (3)	H1C—C1—H1D	109.5
N2—C3—N1	114.4 (2)	N3—C9—C8	123.1 (3)
N2—C3—C4	124.0 (2)	N3—C9—C11	116.5 (3)
N1—C3—C4	121.7 (3)	C8—C9—C11	120.4 (3)
C5—C4—C3	117.3 (3)	C9—C11—H11A	109.5
C5—C4—H4A	121.3	C9—C11—H11B	109.5
C3—C4—H4A	121.3	H11A—C11—H11B	109.5
C4—C5—C6	120.7 (3)	C9—C11—H11C	109.5
C4—C5—H5A	119.7	H11A—C11—H11C	109.5
C6—C5—H5A	119.7	H11B—C11—H11C	109.5
C2—N1—C3	129.4 (2)	O1—C2—N1	124.0 (3)
C2—N1—H1A	115.3	O1—C2—C1	122.8 (3)
C3—N1—H1A	115.3	N1—C2—C1	113.3 (3)
C8—C7—C6	120.1 (3)	C12—C13—H13A	109.5
C8—C7—H7A	120.0	C12—C13—H13B	109.5
C6—C7—H7A	120.0	H13A—C13—H13B	109.5
O2—C12—O3	123.8 (3)	C12—C13—H13C	109.5
O2—C12—C13	123.9 (3)	H13A—C13—H13C	109.5
O3—C12—C13	112.2 (3)	H13B—C13—H13C	109.5
			
C9—N3—C10—N2	−179.5 (3)	C7—C6—C5—C4	179.3 (3)
C9—N3—C10—C6	0.7 (4)	C10—C6—C5—C4	−1.1 (5)
C3—N2—C10—N3	−179.2 (3)	N2—C3—N1—C2	−170.8 (3)
C3—N2—C10—C6	0.6 (4)	C4—C3—N1—C2	10.3 (5)
C5—C6—C10—N3	−179.7 (3)	C5—C6—C7—C8	179.0 (3)
C7—C6—C10—N3	0.0 (4)	C10—C6—C7—C8	−0.6 (5)
C5—C6—C10—N2	0.6 (4)	C6—C7—C8—C9	0.6 (5)
C7—C6—C10—N2	−179.8 (3)	C10—N3—C9—C8	−0.7 (4)
C10—N2—C3—N1	179.9 (3)	C10—N3—C9—C11	179.8 (3)
C10—N2—C3—C4	−1.3 (5)	C7—C8—C9—N3	0.0 (5)
N2—C3—C4—C5	0.8 (5)	C7—C8—C9—C11	179.5 (3)
N1—C3—C4—C5	179.5 (3)	C3—N1—C2—O1	−2.5 (5)
C3—C4—C5—C6	0.5 (5)	C3—N1—C2—C1	177.9 (3)

### Hydrogen-bond geometry (Å, º)

**Table:**

*D*—H···*A*	*D*—H	H···*A*	*D*···*A*	*D*—H···*A*
O3—H3*A*···N2^i^	0.82	1.96	2.774 (3)	173
N1—H1*A*···O2^ii^	0.86	2.07	2.931 (3)	178

Symmetry codes: (i) *x*+1, *y*−1, *z*; (ii) *x*−1, *y*+1, *z*.
